# Gestational thrombocytopenia among pregnant Ghanaian women

**Published:** 2012-06-18

**Authors:** Edeghonghon Olayemi, Frederick William Akuffo

**Affiliations:** 1 Department of Haematology, University of Ghana Medical School, Ghana; 2 Department of Medical Laboratory Sciences, School of Allied Health Sciences, University of Ghana, Ghana

**Keywords:** Pregnancy, thrombocytopenia, Ghana, women, malaria

## Abstract

**Background:**

Thrombocytopenia is a common problem during pregnancy that is not frequently detected and as a result is often inappropriately managed. The obvious concern with thrombocytopenia during pregnancy is the risk of significant bleeding at the time of delivery. This study was designed to determine the prevalence of gestational thrombocytopenia in pregnant women reporting for ante-natal care at a Ghanaian primary health care centre.

**Methods:**

Platelet count was evaluated in 300 blood samples from pregnant women and 100 non pregnant female blood donors. The platelet counts were performed using Sysmex KX-21N automated hematology analyzer. The study design was cross sectional. Proportions were analyzed for statistical significance with the Chi square, Odds ratio was also calculated

**Results:**

The prevalence of thrombocytopenia in pregnant women in this study was 15.3% compared with 4% in controls. This was statistically significant with a P value of 0.003. Odds ratio was 4.31 (95% CI: 1.52-12.04). Most cases of thrombocytopenia were mild (76%), only 4% of the women with thrombocytopenia had severe thrombocytopenia.

**Conclusion:**

The frequency of thrombocytopenia in this study was higher than that reported from more developed parts of the world. This may be due to undetected malaria infection in our patients. Pregnant women should be routinely screened for thrombocytopenia. Those found to be thrombocytopenic should have both thick and thin blood films done to exclude the presence of malaria parasites.

## Background

Platelets are non -nucleated cellular fragments of megakaryocytes, they play a critical role in haemostasis [1]. Thrombocytopenia is said to be present when a patient's platelet count is less than 150,000 X 10^9^ / L. The normal reference range for platelets in the non-pregnant woman is 150,000 to 400,000 X 10^9^ / L. Due to haemodilution secondary to expansion of plasma volume, platelet count in normal pregnancies may decrease by approximately 10%, most of this decrease occurs during the third trimester [2–5]; though the absolute platelet count remains within normal reference range in most patients [3, 6]. Thrombocytopenia can be classified as mild (platelet count of 100,000-150,000 X 10^9^/L), moderate (platelet count of 50,000-100,000 X 10^9^/L) or severe (platelet count less than 50,000 X 10^9^/L) [1].

In pregnancy, most cases are due to gestational thrombocytopenia, idiopathic thrombocytopenic purpura or preeclampsia [7]. Other causes include infections such as malaria, folate deficiency, and diseases such as leukaemia and aplastic anemia [4].

According to Ruggeri et al “ Gestational thrombocytopenia (GT) is characterized by incidental detection of mild to moderate reduction in platelet count during pregnancy in otherwise healthy women with no previous history of idiopathic thrombocytopenia purpura (ITP) or conditions known to be associated with thrombocytopenia”. It is not an early manifestation of autoimmune disease, there is no significant fetal or maternal morbidity and normalization of platelet counts occur in the vast majority of patients post partum [8]. The incidence of thrombocytopenia in neonates born to GT patients is similar to those reported in non- GT women [7, 9, 10].

In a study by Burrows and Kelton, gestational thrombocytopenia (GT) was responsible for about 75% of thrombocytopenia in pregnant women [7]. Gestational thrombocytopenia is usually mild and is not usually associated with fetal thrombocytopenia [11].

Platelet count in patients with GT is usually above 110,000 X 10^9^/L, though counts as low as 70,000 X 10^9^/L have been reported. In patients with counts less than 70,000 X 10^9^/L an alternative explanation is frequently present. Though the pathogenesis of gestational thrombocytopenia is not well understood, it may involve factors such as haemodilution and/or accelerated platelet clearance [12]. Confirmation of a normal platelet count prior to pregnancy decreases the probability of underlying immune thrombocytopenic purpura.

It is known that pregnant women with thrombocytopenia have a higher risk of bleeding excessively during or after childbirth, particularly if they need to have a caesarean section or other surgical intervention during pregnancy, labour or in the peuperium. Such bleeding complications are more likely when the platelet count is less than 50 X 10^9^/L [1].

This study was designed to determine the prevalence of gestational thrombocytopenia among pregnant women reporting for antenatal care at a primary health care centre in Ghana.

## Methods

### Study Population

This was a cross sectional study of pregnant women attending the Mamprobi polyclinic antenatal clinic. During the study period all pregnant women who gave informed consent and met the study inclusion criteria were included in the study.

### Inclusion Criteria

Among the pregnant women who gave informed consent, only those who were normotensive with blood pressure less than or equal to 140/ 90 mmHg) and those without any malaria parasites or platelet aggregation seen on peripheral film examination were included.

### Exclusion Criteria

Pregnant women with the following conditions were excluded from the study: Bleeding disorders, Women on non-steroidal anti-inflammatory drugs such as aspirin, Splenomegaly, Connective tissue disease such as SLE, Hypertension, HIV and hepatitis B infection. Information such as drug history, presence of splenomegaly and HIV / hepatitis B status were extracted from the clinical notes.

### Subjects

A total of three hundred consecutive pregnant women who gave informed consent were selected over a 3 month period from the Mamprobi Polyclinic antenatal care unit. These were subjects who had passed the screening criteria. Data collected from them was entered into a data sheet. The control subjects consisted of 100 age-matched non-pregnant healthy female blood donors.

### Ethics

The research was approved by the Ethics Review Committee of the School of Allied Health Sciences, University of Ghana.

### Sample Collection

Blood specimen was withdrawn with minimal stasis from the ante-cubital vein using a dry sterile disposable syringe and needle. Three milliliters of blood was dispensed into EDTA anticoagulant tubes. The specimens were labeled with subject's age, sex and identification number. The EDTA samples were kept at room temperature until processed within 4hours of collection.

### Laboratory Analysis

Platelet count was performed using the Sysmex KX-21N Automated haematology Analyzer. Standardization, calibration of instrument and processing of samples were done according to manufacturer's instructions.

### Quality Control

Thin blood films stained with Leishman stain were prepared for all blood samples, to confirm thrombocytopenia and exclude the presence of platelet aggregation and malaria parasites.

### Statistical Analysis

Students T test was used to test for the significance between mean platelet counts of pregnant women and controls. Chi- square was used to test for statistical significance between the proportion of pregnant women who were thrombocytopenic and normal controls. The Odds ratio was also calculated. A? value less than 0.05 was considered significant.

## Results

A total of three hundred pregnant women and 100 non- pregnant controls were recruited. Forty-six (15.3%) pregnant women were thrombocytopenic, compared with four (4%) control subjects (p = 0.003); Odds ratio: 4.31 (95% confidence interval 1.5235 to 12.04004).

**Figure 1:**
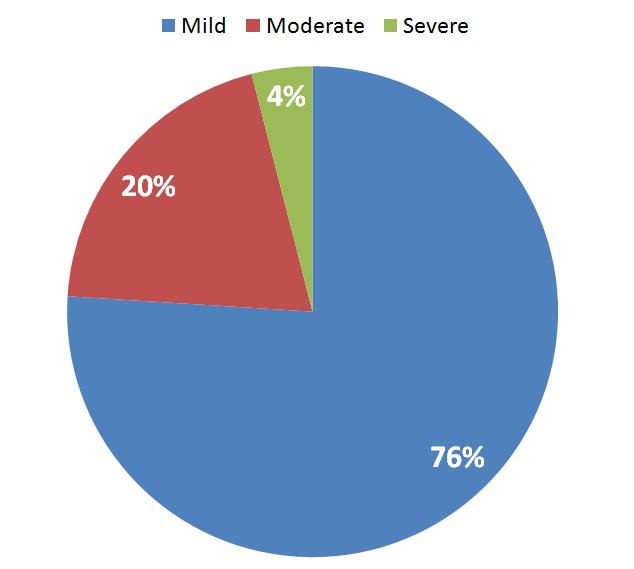
Severity of thrombocytopenia among the pregnant women

Figure 1 illustrates the severity of thrombocytopenia among pregnant women. Out of the 46 pregnant women who were thrombocytopenic most of them 76% had mild thrombocytopenia. However, all the thrombocytopenic control subjects had mild thrombocytopenia.

**Figure 2:**
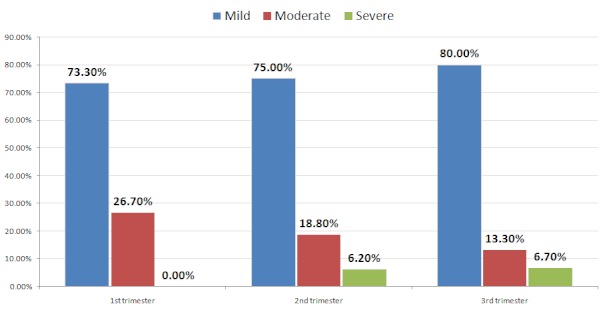
Distribution of thrombocytopenia among pregnant women at different trimesters

Figure 2 shows the distribution of thrombocytopenia among pregnant women at different trimesters. In the first trimester, 73.3% of pregnant women who were thrombocytopenic had mild thrombocytopenia, compared with 75% and 80% in the second and third trimesters respectively. No pregnant woman in the first trimester had severe thrombocytopenia. Comparison of the proportion of thrombocytopenic women in different trimesters, gave a p value of 0.46. Thus there was no significant difference between the proportion of women who were thrombocytopenic in the first trimester and the second and third trimester.

However, there was significant difference between the proportion of pregnant thrombocytopenic women and non-pregnant controls, P value of 0.003.

## Discussion

The present work was designed to determine the prevalence of gestational thrombocytopenia in pregnant women attending antenatal care at a Ghanaian primary health care centre. Significantly more pregnant women in this study were thrombocytopenic compared with non-pregnant healthy controls and pregnant women were at least four times more likely to be thrombocytopenic compared to controls.

The prevalence of gestational thrombocytopenia in our study was 15.3%. This figure was higher than figures of 11.6% reported by Boehlen et al in 2006 [6] and 7.2% reported by Sainio et al in 2000 [2].

The higher prevalence in this study may be as a result of malaria infection. Clerk et al in 2009 reported that the prevalence of malaria among pregnant women in Ghana was 47% [13]. Immunity is known to be reduced in pregnancy and pregnant women are prone to malaria infection. Though pregnant women in this study were screened for the presence of malaria and those who had malaria parasites on thin blood film examination were excluded. The sensitivity of the thin blood film in detecting malaria parasites is dependent on the parasite load, so it is not impossible that some of the pregnant women may have asymptomatic parasitaemia which was missed.

Again, Sainio et al [2] only studied full term pregnant women so they might have missed some pregnant women who were thrombocytopenic earlier in pregnancy. Thus, the fact that this study included pregnant women in all trimesters may explain the higher prevalence.

From our findings, gestational thrombocytopenia occurred across the three trimesters, this was against the report of Crowther et al (1996) [14] who reported that gestational thrombocytopenia in pregnancy is a disorder that develops primarily in the late second or third trimester.

Most of the cases of thrombocytopenia (76%) in our study were mild with platelet counts above; this agrees with the finding of Boehlen (2006) [6] who reported that gestational thrombocytopenia is usually mild.

## Conclusion

The prevalence of thrombocytopenia in this study was 15.3%. This rather high figure could be as a result of malaria infection which is prevalent among pregnant women in this environment. With the relatively higher prevalence of thrombocytopenia found in this study, pregnant women should be screened for thrombocytopenia. Those found to be thrombocytopenic should have both thick and thin blood films done to exclude the presence of malaria parasites. It may also be useful to investigate further the effectiveness of current malaria prophylaxis used in the current to exclude the possibility of resistance.

Since gestational thrombocytopenia is usually mild or moderate, pregnant women found to have severe thrombocytopenia should be investigated to exclude other causes of thrombocytopenia in pregnancy such as autoimmune diseases and pre-eclampsia. Care should be taken during delivery of women with thrombocytopenia especially if severe to avoid bleeding complications. Similarly, children born to thrombocytopenic mothers should also be followed up and any who is found to be thrombocytopenic managed appropriately.
